# Dynamic Rendition of Adipose Genes Under Epigenetic Regulation: Revealing New Mechanisms of Obesity Occurrence

**DOI:** 10.3390/cimb47070540

**Published:** 2025-07-11

**Authors:** Weijing Wen, Simeng Gu, Fanjia Guo, Zhijian Chen, Sujun Yan, Zhe Mo

**Affiliations:** 1 Zhejiang Provincial Center for Disease Control and Prevention, 3399 Bin Sheng Road, Binjiang District, Hangzhou 310051, China; wwwenjing27@outlook.com (W.W.); smgu@cdc.zj.cn (S.G.); gfjia@cdc.zj.cn (F.G.); zhjchen@cdc.zj.cn (Z.C.); 2 School of Public Health, Health Science Center, Ningbo University, Ningbo 315211, China

**Keywords:** epigenetic, DNA methylation, RNA methylation, histone modifications, obesity, adipogenesis, energy metabolism

## Abstract

Obesity is a chronic metabolic disorder and a growing global public health challenge, affecting hundreds of millions of individuals worldwide. While diet and physical activity are well-established contributors, increasing evidence underscores the critical role of epigenetic mechanisms in mediating obesity-related processes. Epigenetic modifications—such as DNA methylation, RNA methylation (particularly N6-methyladenosine), histone modifications, non-coding RNAs, and chromatin remodeling—modulate gene expression without altering the DNA sequence. This review aims to provide an overview of the epigenetic mechanisms involved in obesity, with an emphasis on their molecular functions and regulatory networks. Integrating findings from relevant studies, we discuss how these modifications influence obesity-related outcomes through regulating key processes such as adipocyte differentiation and energy metabolism. Advancing our understanding of epigenetic regulation may pave the way for novel, targeted strategies in the prevention and treatment of obesity.

## 1. Introduction

Obesity is an increasingly severe global public health issue. According to a 2022 report by the World Health Organization (WHO), obesity affects approximately one-eighth of the worldwide population [1]. Beyond its impact on individual quality of life, obesity is closely associated with various metabolic diseases, including type 2 diabetes, metabolic-associated fatty liver disease, hypertension, and multiple malignancies such as endometrial and colorectal cancer [2,3,4]. Fundamentally, obesity results from a chronic imbalance between energy intake and expenditure [5]. A key factor in this process is the heterogeneity of adipose tissue: brown adipose tissue (BAT) and beige adipose tissue contribute to energy expenditure through thermogenesis, whereas white adipose tissue (WAT) regulates energy homeostasis by storing and releasing fatty acids [6]. These adipose tissue types affect the metabolic process of excessive fat accumulation by regulating the dynamic balance between energy consumption and storage [7].

Obesity is a complex condition influenced by genetic and environmental factors [8]. Genetic factors establish the basis for an individual’s susceptibility to obesity, while environmental elements such as diet and physical activity directly impact the development of obesity [9]. Epigenetics—defined as heritable changes in gene function that occur without alterations to the DNA sequence—serves as a critical interface between environmental inputs and gene regulation [10,11,12]. The field of epigenetics has gained increasing prominence for its role in modulating gene expression through mechanisms such as DNA methylation, RNA methylation, and histone modifications [11]. Growing evidence supports the involvement of epigenetic dysregulation in obesity [12,13]. In obese individuals, abnormal histone modifications and alterations in DNA methylation levels are closely linked to key pathological mechanisms such as adipose tissue dysfunction, insulin resistance (IR), and chronic inflammation [14,15]. Moreover, interventions like bariatric surgery have been shown to reverse certain epigenetic modifications, suggesting their potential as therapeutic targets [16].

Among the various epigenetic mechanisms, DNA methylation, RNA methylation (particularly N6-methyladenosine (m^6^A)), histone modifications, non-coding RNAs, and chromatin remodeling have emerged as crucial regulators of adipose tissue development and energy metabolism. This review summarizes their regulatory mechanisms on adipose tissue development and energy metabolism. A deeper understanding of these mechanisms may facilitate the identification of early diagnostic biomarkers and the development of innovative epigenetic therapies for obesity.

## 2. Epigenetic Regulation of Obesity and Underlying Mechanisms

### 2.1. DNA Methylation and Obesity

DNA methylation is an epigenetic mechanism involving the transfer of a methyl group at the C5 position of the cytosine to form 5-methylcytosine (5mC) [17,18]. Within the genome, cytosine-phosphate-guanine (CpG) sites often cluster into regions known as CpG islands, which are frequently located in gene promoter regions [19]. Extensive research has demonstrated that DNA methylation modifications related to obesity are mostly concentrated at specific CpG sites. Through comparing obese and non-obese humans, researchers have identified 114 differentially methylated positions (DMPs) in CpG islands [20]. Furthermore, genome-wide DNA methylation analyses of adipocytes from obese individuals versus non-obese individuals have revealed that most altered CpG sites are significantly associated with obesity-related gene expression [21]. Notably, lifestyle interventions such as caloric restriction and bariatric surgery have demonstrated the capacity to partially reverse these methylation alterations, highlighting their potential plasticity and therapeutic relevance [22,23].

DNA methylation is catalyzed by DNA methyltransferases (DNMTs), primarily DNMT1, DNMT3A, and DNMT3B. Three conserved DNMTs are responsible for the de novo establishment and maintenance of DNA methylation patterns [24,25]. Conversely, active DNA demethylation is mediated by the ten-eleven translocation (TET) family of methylcytosine dioxygenases [26]. These methylation marks are interpreted by methyl-binding proteins (MBPs), which recognize methylated CpG sites and recruit chromatin remodeling complexes to activate or repress transcription, depending on the cellular context [27]. In obesity research, overexpression of DNMTs results in excessive methylation of promoter regions in genes regulating lipid metabolism, leading to gene silencing and excessive fat accumulation [28,29]. In contrast, DNMT deficiency can alleviate aberrant methylation in obesity-related genes, restoring their transcriptional activity and mitigating pathological fat deposition [30] (Table 1).

#### 2.1.1. Regulation of DNA Methylation in Adipose Tissue Development and Differentiation

DNMTs play a pivotal role in the regulation of adipose tissue development and differentiation by modulating the methylation status of genes central to adipogenesis and metabolic function. Studies have shown that DNMT1 exhibits stage-specific regulation during the differentiation of 3T3-L1 preadipocytes. In early differentiation, inhibition of DNMT1 reduces DNA methylation at the promoter region of wingless-type MMTV integration site family, member 10a (*Wnt10a*)—a known anti-adipogenic gene—thereby promoting its expression. Overexpression of WNT10A impairs adipogenesis by inhibiting the differentiation of preadipocytes [41]. Conversely, during the late stage of differentiation, DNMT1 inhibition decreases methylation at the sterol regulatory element-binding protein 1c (*Srebp-1c*) promoter, leading to upregulation of SREBP-1C (a pro-lipogenic transcription factor), which enhances lipid synthesis and adipocyte maturation [42,43,44]. Intriguingly, DNMT3A also exhibits tissue-specific roles in adipose tissue. DNMT3A acts as an epigenetic suppressor of insulin sensitivity by methylating the promoter of fibroblast growth factor 21 (*Fgf21*), thereby reducing its expression and promoting IR [45,46]. However, liver-specific knockdown of DNMT3A in mice attenuates fibroblast growth factor 19 (FGF19)-mediated DNA methylation at the fatty acid synthase (*Fasn*) promoter and weakens the inhibition of hepatic lipogenesis [47]. Furthermore, the impact of DNMT3A on adipocyte differentiation is linked to the epithelial–mesenchymal transition (EMT) process. Experiments have shown that DNMT3A deficiency or dysfunction leads to the enrichment of EMT-related genes in adipocytes and reduces the differentiation of adipocytes [48].

DNA methylation modulates adipogenesis by regulating transcription factors. Notably, peroxisome proliferator-activated receptor γ (PPARγ) is a key transcriptional regulator that governs adipocyte differentiation and the expression of lipid metabolism-related genes [49,50]. Treatment with the DNA demethylating agent 5′-azacytidine induces the differentiation of fibroblast NIH-3T3 cells into adipocytes by targeting zinc finger protein 423 (*Zfp423*). Specifically, 5′-azacytidine reduces methylation within the *Zfp423* promoter, which initiates chromatin remodeling into a transcriptionally active conformation, thereby promoting *Zfp423* expression [51,52]. The upregulation of *Zfp423* further enhances the expression of PPARγ2 and differentiation markers, facilitating adipogenesis in NIH-3T3 cells [53].

DNA methylation plays an essential role in regulating brown and beige adipocytes. Uncoupling protein 1 (UCP1) is a mitochondrial membrane protein and is involved in adipocyte thermogenesis [54,55]. Obesogenic diets specifically regulate visceral adipose tissue-derived serine protease inhibitor (VASPIN) expression in BAT, a process associated with DNA methylation changes. VASPIN expression correlates positively with *Ucp1* expression [56]. However, the specific molecular mechanism by which VASPIN regulates BAT function remains unclear. Beyond DNMT, recent studies have revealed additional roles of DNA-modifying enzymes. The DNA demethylase ten-eleven translocation 1 (TET1) overexpression impairs the thermogenic capacity of beige adipocytes and contributes to obesity development. Interestingly, this effect is largely DNA demethylase-independent. Instead, TET1 interacts with histone deacetylase 1 (HDAC1) to mediate epigenetic changes that suppress the expression of key thermogenic genes (such as *Ucp1* and *Ppargc1a*) [57]. In summary, DNA methylation regulates multiple stages of adipogenesis, but further exploration is needed to fully elucidate the complex interactions and tissue-specific effects.

#### 2.1.2. DNA Methylation and Obesity-Related Metabolism

DNA methylation plays a pivotal role in regulating metabolic homeostasis and energy balance. Adiponectin, an adipocyte-derived hormone, enhances metabolic homeostasis through AMP-activated protein kinase (AMPK) and stimulates fatty acid oxidation via peroxisome proliferator-activated receptor α (PPARα) [58,59]. In contrast, resistin is an adipose-derived hormone that promotes inflammation and IR, ultimately disrupting energy homeostasis [60]. It has been demonstrated that antibiotic exposure alters the gut microbiota in obese mice, resulting in reduced expression of DNMT1 and DNMT3A. These modifications increase mRNA expression of adiponectin and resistin while activating key metabolic pathways and suppressing lipid accumulation [61]. Mechanistically, DNMT1 overexpression in adipocytes induces hypermethylation of a specific region (R2) within the adiponectin promoter, thereby suppressing adiponectin expression and promoting adipogenesis [62,63]. Moreover, a high-fat diet (HFD) augments the stability of DNMT1 through a ubiquitination-mediated mechanism. Liver-specific deletion of either *Dnmt1* or *Dnmt3a* upregulates Beta-klotho expression and fatty acid oxidation, ultimately ameliorating HFD-induced hepatic steatosis [64].

DNA methylation also directly impacts glucose metabolism through the regulation of insulin signaling. The insulin receptor (INSR) is a critical mediator of insulin-dependent glucose uptake. Hypermethylation of the *Insr* promoter has been shown to repress gene expression, impairing the signaling pathway and consequently disrupting glucose uptake and utilization [65,66]. Clinical investigations have demonstrated a notable inverse correlation between the levels of INSR in adipose tissue and the body mass index (BMI) [67,68]. In addition, MBPs critically modulate metabolic outcomes. Methyl-CpG binding domain protein 2 (MBD2) acts as a reader of DNA methylation and influences the transcription of genes involved in energy expenditure and storage. Consequently, *Mbd2*-knockout mice exhibit enhanced glucose utilization, increased metabolic flexibility, and resistance to the deleterious effects of HFD, highlighting the physiological significance of methylation-dependent transcriptional regulation [69]. These studies have identified DNA methylation markers associated with metabolic regulation. Nevertheless, emerging evidence suggests that certain methylation changes may be reversible through lifestyle interventions such as dietary modification and physical activity [70,71]. However, the underlying mechanisms mediating these effects remain poorly understood.

#### 2.1.3. DNA Methylation in the Adipose Tissue Microenvironment

DNA methylation plays a key role in regulating pathological processes within the adipose tissue microenvironment. Recent evidence indicates that obesity-derived macrophages can induce apoptosis of glial cells by upregulating tumor necrosis factor α (TNF-α) and activating the nuclear factor kappa-light-chain-enhancer of activated B cells (NF-κB)/PH domain leucine-rich repeat protein phosphatase 1 (PHLPP1) axis, thereby contributing to adipose tissue inflammation [72]. Among CD4^+^ T cells, the methylation levels of 79 CpG sites are strongly correlated with the amount of visceral adipose tissue, including 4 CpG sites in the promoter region of calsyntenin 1 (*Clstn1*) [73]. Moreover, in subcutaneous adipose tissue and circulating leukocytes, the methylation levels of fibroblast growth factor receptor-like 1 (*Fgfrl1*), non-SMC condensin II complex subunit H2 (*Ncaph2*), paroxysmal nonkinesigenic dyskinesia (*Pnkd*), and SMAD family member 3 (*Smad3*) exhibit strong and statistically significant efficiencies in distinguishing obesity from non-obese status, as well as a great correlation between both tissues [74]. In macrophages, obesity-related factors alter DNA methylation of the *Pparγ1* promoter, suppressing PPARγ1 expression and contributing to the establishment of a pro-inflammatory phenotype [75].

### 2.2. RNA Methylation and Obesity

To date, over 170 RNA modifications have been identified across all classes of RNA molecules [76]. Among these, RNA methylation has emerged as a fundamental post-transcriptional regulatory mechanism, critically involved in diverse aspects of RNA metabolism, including splicing, stability, nuclear export, localization, and translation efficiency [77,78]. Among the various types of RNA modifications, N6-methyladenosine (m^6^A) has been the most extensively studied [79,80,81]. Additionally, modifications like N1-methyladenosine (m^1^A), 5-methylcytosine (m^5^C), and 7-methylguanosine (m^7^G) have also gained increasing attention in recent years. Given the central role of m^6^A in previous research, this review exclusively focuses on m^6^A modification.

The m^6^A modification is a dynamic and reversible co-transcriptional process involving three key components: methyltransferases, demethylases, and m^6^A-binding proteins [82] (Table 2). Methyltransferases act as “Writers” by catalyzing RNA methylation to generate m^6^A modification [83]. The m^6^A methyltransferase complex comprises functionally distinct components organized into two subcomplexes: the m^6^A-Methyltransferase-like (METTL) complex (MAC) and the m^6^A–METTL–associated complex (MACOM) [84,85]. More specifically, METTL3 and METTL14 are the core components of MAC [86]. METTL3 provides methyltransferase activity [87,88], while METTL14 plays a structural role in stabilizing the complex and facilitating RNA binding [89]. MACOM includes proteins such as Wilms tumor 1-associated protein (WTAP), E3 ubiquitin ligase Hakai, and vir-like m^6^A methyltransferase-associated protein (VIRMA/KIAA1429) [90,91]. Although MACOM lacks intrinsic catalytic activity, its components interact with the METTL3-METTL14 heterodimer to ensure the efficient and precise m^6^A methylation [92,93]. Demethylases, termed “Erasers,” reverse m^6^A modifications and thereby restore RNA to its unmethylated state [94]. Among them, the fat mass and obesity-associated protein (FTO) is a well-characterized m^6^A demethylase that exhibits a preference for N6,2′-O-dimethyladenosine (m^6^A_m_) located at the 5′mRNA cap structure [95,96]. Compared to m^6^A, m^6^A_m_ contains an additional 2′-O-methyl group on the ribose moiety, and FTO demethylates m^6^A_m_ with higher efficiency, which leads to decreased stability of m^6^A_m_ mRNAs [97,98]. Another critical demethylase is AlkB homolog 5 (ALKBH5), which specifically catalyzes the demethylation of m^6^A to adenosine, demonstrating greater substrate specificity and catalytic efficiency [99]. In addition to its demethylase activity, ALKBH5 also plays a distinct role in regulating mRNA export within nuclear speckles [100]. m^6^A-binding proteins, known as “Readers”, interpret m^6^A modifications by recognizing methylated mRNAs and mediate downstream biological processes [101]. YTH domain family proteins influence the fate of modified RNAs by modulating processes such as splicing, stability, and degradation [102]. For example, YTH domain-containing family protein 1 (YTHDF1) promotes the translation of m^6^A-modified mRNA, whereas YTHDF2 facilitates their degradation [103]. In contrast to the degradation-promoting function of YTHDF2, insulin-like growth factor 2 mRNA-binding proteins (IGF2BP1–3) promote the stabilization of target mRNAs in an m^6^A-dependent manner, thereby affecting gene expression output under normal and heat shock conditions [104].

In a differential gene expression analysis comparing lean and obese individuals, WTAP and VIRMA are identified as significantly upregulated in adipose tissues. In addition to these MACOM components, other m^6^A regulatory proteins have been implicated in obesity. For example, the expression levels of ALKBH5 and YTHDF3 in peripheral blood mononuclear cells (PBMCs) correlate with obesity [105]. Notably, *Fto* mRNA expression is markedly elevated in obese individuals and shows a strong positive association with BMI and other obesity-related indicators [106,107].

#### 2.2.1. Regulation of RNA Methylation in Adipose Tissue Development and Differentiation

m^6^A modification regulates adipogenesis through methyltransferases, demethylases, and m^6^A-binding proteins. Notably, METTL3 has been extensively linked to obesity. In male mice subjected to a long-term diet-induced obesity model, *Mettl3* mRNA expression is reduced [108]. Similarly, during the differentiation of porcine intramuscular adipocytes, dynamic changes in m^6^A levels are primarily attributed to altered expression of METTL3 and METTL14 [109]. Functionally, METTL3 facilitates WAT beiging by enhancing the stability of thermogenic mRNAs, including Krüppel-like factor 9 (*Klf9*) [110]. In BAT, prostaglandin E receptor 3 (EP3) has been shown to maintain WTAP expression via activation of the protein kinase A (PKA)–extracellular signal-regulated kinase 1/2 (Erk1/2) signaling pathway. Loss of *Ep3* inhibits WTAP-mediated m^6^A methylation, resulting in downregulation of zinc finger protein 410 (*Zfp410*), ultimately impeding the BAT differentiation program [111]. In parallel, demethylation pathways are involved in adipogenesis. Genes regulated by the nicotinamide adenine dinucleotide phosphate (NADP)/FTO axis are mainly involved in the modulation of white adipocyte differentiation and chromatin remodeling enzymes. The knockdown of *Fto* blocks NADP-enhanced adipogenesis in 3T3-L1 preadipocytes [112]. Moreover, m^6^A-binding proteins play a pivotal role in post-transcriptional regulation during adipose tissue remodeling. For instance, YTHDF1 enhances the translation of bone morphogenetic protein 8b (*Bmp8b*) mRNA by specifically binding to its 3′ untranslated region (3′UTR), thereby facilitating inguinal WAT beiging and alleviating obesity [113].

These three categories of proteins can act in concert to regulate adipogenesis, especially through the FTO-YTHDF2 axis. At the cellular cycle level, FTO promotes cyclin-dependent kinase 2 (CDK2) and cyclin A2 (CCNA2) expression via its m^6^A demethylation activity, thus driving cell cycle progression [114]. Conversely, YTHDF2 recognizes and promotes the decay of methylated *Cdk2* and *Ccna2* mRNAs, blocking the mitotic clonal expansion and inhibiting adipogenesis [115,116]. In terms of cellular differentiation, silencing of *Fto* leads to enhanced methylation of Janus kinase 2 (*Jak2*) mRNA, which in turn promotes YTHDF2-mediated degradation of *Jak2* transcripts. This results in the inactivation of JAK2–signal transducer and activator of transcription 3 (STAT3) signaling and subsequent downregulation of CCAAT/enhancer-binding protein β (C/EBPβ), ultimately inhibiting adipogenesis [117]. Notably, this regulatory axis also influences autophagy. Upon *Fto* silencing, YTHDF2 targets highly methylated autophagy-related 5 (*Atg5*) and autophagy-related 7 (*Atg7*) mRNA, causing their degradation and reduced protein expression, thereby impairing autophagic activity and further suppressing adipogenesis [118].

In addition to the aforementioned m^6^A modification, modifications of m^5^C on mRNAs have been identified in adipogenesis-related studies. NOP2/Sun domain family, member 2 (NSUN2) catalyzes the m^5^C modification of cyclin-dependent kinase inhibitor 1a (*Cdkn1a*) mRNA. Aly/REF export factor (ALYREF) is an m^5^C reader protein that binds to the modified *Cdkn1a* and facilitates its transportation from the nucleus to the cytoplasm. This enhancement in cytoplasmic localization facilitates increased translation efficiency of *Cdkn1a*, leading to increased protein expression, which subsequently suppresses the cell cycle and reduces adipogenesis [119]. Similarly, ALYREF recognizes and exports Y-box binding protein 2 (*Ybx2*) and smoothened, frizzled class receptor (*Smo*) mRNAs with m^5^C modifications, resulting in elevated protein expression of YBX2 and SMO, which inhibit adipogenesis and promote myogenesis, respectively [120]. Moreover, recent evidence indicates that YBX1 regulates the stability of m^5^C-modified mRNAs involved in autophagy and ubiquitination pathways, such as unc-51-like kinase 2 (*Ulk2*) and *Ulk1*, thus enhancing autophagy and adipogenesis [121,122]. Collectively, these findings suggest that RNA methylation regulates adipogenesis by orchestrating key biological processes such as cell cycle progression, cellular differentiation, and autophagy.

#### 2.2.2. RNA Methylation and Obesity-Related Metabolism

The m^6^A modification plays a vital role in the regulation of lipid metabolism. Recent studies have demonstrated that mice with liver-specific knockout of *Mettl3* exhibit significant hepatic lipid accumulation and elevated serum total cholesterol levels after four weeks. Gene set enrichment analysis further reveals abnormal activation of lipid metabolism pathways [123], indicating that METTL3 functions as a key regulator of lipid homeostasis. Specifically, METTL3 overexpression increases the methylated level of *Fasn* mRNA, subsequently facilitating fatty acid metabolism [124]. In addition, METTL3 directly binds to *Rubicon* mRNA and mediates its m^6^A modification, which in turn modulates lipid metabolism by regulating autophagic activity [125].

The role of m^6^A modification in glucose metabolism is indispensable. Several studies have demonstrated that m^6^A modification influences the stability and translation of key glycolytic enzyme mRNAs to modulate glycolytic activity [126,127]. In beige adipocytes, METTL3 and IGF2BP2 cooperatively enhance the stability of glycolysis-related mRNAs, promoting glucose uptake and metabolic activity. This process also increases lactate production, which in turn facilitates preadipocyte proliferation [128]. Moreover, m^6^A modification intersects with key metabolic signaling pathways. Leptin activates the JAK-STAT signaling pathway in the hypothalamus to regulate insulin sensitivity [129]. However, overexpression of FTO in liver cells impairs the leptin-induced phosphorylation process of STAT3, resulting in the disruption of glucose metabolism [130]. Conversely, ALKBH5 enhances the stability of glucagon receptor (*Gcgr*) mRNA through its demethylase activity, leading to elevated blood glucose levels [131]. Mechanistically, GCGR, located on the surface of hepatocytes, binds to glucagon and promotes hepatic gluconeogenesis by promoting cyclic adenosine monophosphate (cAMP)-PKA-dependent activation of the cAMP response element-binding protein (CREB)-CREB-binding protein (CBP)-CREB-regulated transcription coactivator 2 (CRTC2) complex [132,133]. Taken together, these findings highlight that m^6^A modification exerts a crucial role in energy metabolism through the regulation of key metabolic genes and signaling pathways.

**Table 2:** The functions of m^6^A-related protein.

Key Components	Enzymes Included	Functions	Target RNAs	Obesity-Related Functions	References
Methyltransferases (Writer)	METTL3, METTL14, etc.	Catalyze the methylation of specific adenosines in RNA	*Fasn*, *Rubicon*, *Klf9*, glycolysis-related mRNAs	Regulate lipid metabolism, promote WAT beiging, modulate autophagy, enhance glycolysis and glucose uptake	[108,109,110,111,124,125,128]
Demethylases (Eraser)	FTO, ALKBH5, etc.	Removal of m^6^A methylation modification on RNA	*Gcgr*, *Jak2*, *Atg5*, *Atg7*, *Cdk2*, *Ccna2*	Modulate gluconeogenesis, autophagy, insulin sensitivity, cell cycle, adipocyte differentiation	[112,114,117,118,130,131]
m^6^A-binding proteins (Reader)	YTH domain family proteins, IGF2BP1–3, etc.	Recognizes and binds RNAs with m^6^A and participates in the regulation of RNA metabolism	*Bmp8b*, *Jak2*, *Atg5*, *Atg7*, *Cdk2*, *Ccna2*, glycolytic enzyme mRNAs	Promote adipogenesis, regulate cell cycle, glucose metabolism, RNA stability, thermogenesis in adipose tissues	[113,115,116,117,118,128]

The columns represent the following: Key Components, functional categories of m^6^A regulators (writers, erasers, readers); Enzymes Included, representative enzymes in each category; Functions, main roles in RNA methylation; Target RNAs, example transcripts affected by these enzymes; Obesity-related Functions, related roles in adipose tissue biology and obesity; References, citation number corresponding to each study, as listed in the reference section. Abbreviations: *Fasn*, fatty acid synthase; *Klf9*, Krüppel-like factor 9; *Gcgr*, glucagon receptor; *Jak2*, janus kinase 2; *Atg5*, autophagy-related 5; *Cdk2*, cyclin-dependent kinase 2; *Ccna2*, cyclin A2.

Although direct involvement of m^1^A modification in adipocyte development and metabolic regulation has not yet been established, it is broadly distributed across various types of RNA. Its enrichment on tRNAs and the impact they exert on tRNA structural dynamics suggest a possible role in modulating adiposity-related biological processes [85,134,135]. Supporting this possibility, hepatic transcriptomic m^1^A levels were found to be elevated in a mouse model of non-alcoholic steatohepatitis (NASH), accompanied by increased expression of YTH family reader proteins [136]. Additionally, the m^1^A demethylase ALKBH3 has been shown to enhance glycolysis in cancer cells by modulating the expression of m^1^A-modified ATP synthase subunit delta (*Atp5d*) mRNA [137]. In addition, NOP2/Sun RNA methyltransferase family member 3 (NSUN3)-dependent RNA modifications—m^5^C and its derivative 5-formylcytosine (f^5^C)—enhance mitochondrial mRNA translation [138]. When mitochondrial m^5^C levels are reduced, oxidative phosphorylation declines, and glycolysis is upregulated to compensate for the energy deficit [139]. In another mechanism, ALYREF stabilizes pyruvate kinase M2 (*Pkm2*) by binding to its m^5^C sites in the 3′UTR, leading to enhanced glycolysis [140]. These findings imply that RNA modifications like m^1^A and m^5^C regulate energy metabolism, though their roles in obesity and metabolic diseases remain to be clarified.

#### 2.2.3. RNA Methylation in the Adipose Tissue Microenvironment

Beyond adipocytes, m^6^A modification also plays pivotal roles in other cell types within the adipose tissue microenvironment. Emerging studies demonstrate that myeloid lineage–restricted deletion of METTL3 prevents obesity in mice with improved inflammatory and metabolic phenotypes. At the mechanistic level, loss of METTL3 results in a marked upregulation of DNA damage inducible transcript 4 (*Ddit4*) mRNA, a key regulator of metabolic adaptation in macrophages. Elevated DDIT4 expression inhibits mammalian target of rapamycin (mTOR) signaling and modulates effector functions of macrophages, promoting an anti-inflammatory phenotype [141]. Consistently, IGF2BP2 switches M1 macrophages to M2 activation by targeting tuberous sclerosis 1 via an m^6^A-dependent manner [142]. Furthermore, FTO-related m^6^A modification regulates microglia-induced inflammatory responses by stabilizing a disintegrin and metalloprotease 17 (*Adam17*) mRNA expression [143]. These findings provide new insights into the multicellular regulatory mechanisms of m^6^A modification within the adipose tissue microenvironment.

### 2.3. Histone Modifications and Obesity

The histone family consists of H1, H2A, H2B, H3, and H4. Among them, two copies each of the core histones—H2A, H2B, H3, and H4—assemble to form the nucleosome core particle [144,145]. In contrast, H1 binds to the DNA entry and exit sites on the nucleosome, thereby contributing to the stabilization of higher-order chromatin structure [146,147]. Histone modifications refer to biochemical processes in which histone-modifying enzymes add or remove chemical groups to specific amino acid residues on histone and non-histone proteins [148]. These modifications can influence gene expression in two main ways: by altering chromatin structure and by recruiting regulatory factors [149,150]. Common types of histone modifications include phosphorylation, acetylation, SUMOylation, methylation, and ubiquitination [151]. While each histone modification operates through a distinct mechanism, they also exhibit interdependence and collectively participate in the regulation of multiple biological processes [152,153].

Obesity can alter adipose histone modifications, affecting adipocyte development and function [154]. Numerous studies have established a strong association between histone modifications and obesity, as well as their involvement in intergenerational epigenetic inheritance. Compared with the control group, the histone methylation level (H3K4me3) of lipid metabolism genes is dramatically elevated in obese Caenorhabditis elegans [155]. Similarly, in mammals, obese mice exhibit significant differences in histone modifications compared to normal-weight counterparts, including alterations in arginine methylation and lysine methylation [156]. Furthermore, histone modifications are a potential mechanism for intergenerational and transgenerational epigenetic effects [157]. In HFD-induced obese mice, altered levels of histone methylation and acetylation have been observed in germ cells of both sexes [158]. Further studies have identified that maternal mice fed an HFD alter epigenetic marks (H3K27me3 and H3K27ac) of numerous genes in the bone tissue of their offspring [159].

#### 2.3.1. Regulation of Histone Modifications in Adipose Tissue Development and Differentiation

Histone modifications interact with adipogenic transcription factors to establish an adipogenic regulatory network. C/EBPβ is expressed during the early stages of adipogenesis and participates in the induction of PPARγ [160]. Histone-modifying enzymes can regulate C/EBPβ expression through diverse mechanisms. Silent mating-type information regulation 2 homolog 1 (SIRT1) suppresses the acetylation of C/EBPβ at lysine 39, as well as histone H3 at lysines 27 (H3K27) and 9 (H3K9), resulting in impaired transcription activity of C/EBPβ [161]. In contrast, protein arginine N-methyltransferase 1 (PRMT1) reduces the expression of Smad ubiquitination regulatory factor 2 (SMURF2), preventing the ubiquitination and degradation of C/EBPβ [162]. At later stages of differentiation, PPARγ and CCAAT/enhancer binding protein α (C/EBPα) are co-expressed and act synergistically to activate numerous key metabolic adipocyte genes [163]. However, this synergistic effect is subject to modulation by histone modifications. The knockdown of nuclear receptor-binding SET domain 2 (*Nsd2*) increases trimethylation of H3K27 (H3K27me3) levels in adipocytes. This elevation of H3K27me3 significantly inhibits C/EBPα and other targets of PPARγ, thus hindering adipogenesis [164]. In addition, phosphorylation of histone H2B at serine 36 (H2B-Ser36p), an AMPK-mediated epigenetic mark, promotes adipocyte precursor differentiation, as evidenced by increased H2B-Ser36p levels during the peak expression of proadipogenic genes [165].

**Figure 1:**
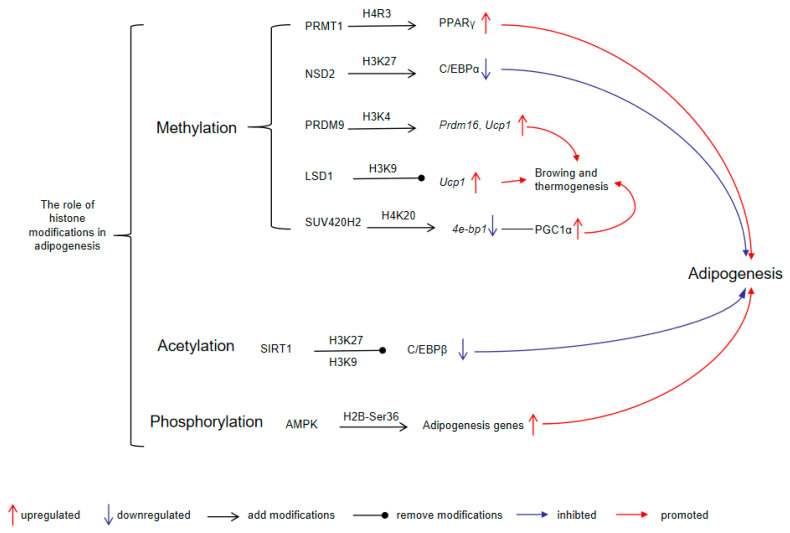
The role of histone modifications in adipogenesis. PRMT1-mediated H4R3 methylation promotes PPARγ expression and adipogenesis; NSD2-mediated H3K27 methylation suppresses C/EBPα expression and inhibits adipogenesis; PRDM9 methylates H3K4 to activate *Prdm16* and *Ucp1*, thereby enhancing browning and thermogenesis; LSD1, a demethylase, removes H3K9 methylation, facilitating *Ucp1* expression; SUV420H2 catalyzes H4K20 methylation, suppressing *4e-bp1* and thereby activating PGC1α, contributing to thermogenic activation and adipogenesis; SIRT1, by deacetylating H3K27 and H3K9, represses C/EBPβ, negatively regulating adipogenesis; AMPK phosphorylates H2B at Ser36, resulting in increased transcription of adipogenesis-related genes. Abbreviations: PRMT1, protein arginine N-methyltransferase 1; NSD2, nuclear receptor-binding SET domain 2; PRDM9, PR domain-containing protein 9; LSD1, lysine-specific demethylase 1; SUV420H2, suppressor of variegation 4-20 homolog 2; SIRT1, silent mating-type information regulation 2 homolog 1; AMPK, AMP-activated protein kinase; PPARγ, peroxisome proliferator-activated receptor γ; C/EBPα, CCAAT/enhancer binding protein α; *Prdm16*, PR domain-containing protein 16; *Ucp1*, uncoupling protein 1; *4e-bp1*, 4E-binding protein 1; PGC1α, peroxisome proliferator-activated receptor-γ coactivator 1α; C/EBPβ, CCAAT/enhancer-binding protein β.

Histone modifications play a critical role in thermogenic adipocyte differentiation and thermogenesis. During the differentiation process of BAT adipocytes, lysine methyltransferase PR domain-containing protein 9 (PRDM9) expression is markedly upregulated [166]. PRDM9 catalyzes the trimethylation of lysine residues 4 and 36 on histone H3 (H3K4me3 and H3K36me3) via its PR/SET domain [167]. Suppression of PRDM9 reduces the binding of H3K4me3 to pivotal genes (such as *Ppargc1b*, *Prdm16*, *Cidea*, and *Ucp1*), which impedes the differentiation of BAT adipocytes [166]. Additionally, through interaction with the transcription factor zinc finger protein 516 (ZFP516), lysine-specific demethylase 1 (LSD1) is recruited to BAT-enriched genes, where it removes methyl groups from H3K9, thus initiating the BAT program and thermogenesis [168]. Furthermore, the histone methyltransferase suppressor of variegation 4-20 homolog 2 (SUV420H2) is essential for brown and beige adipocyte development. It catalyzes trimethylation of lysine 20 on histone H4 (H4K20me3) at the promoter of 4E-binding protein 1 (*4e-bp1*), repressing its expression and thereby increasing peroxisome proliferator-activated receptor-γ coactivator 1α (PGC1α) protein levels, which enhances the thermogenic program [169]. These findings underscore the intricate interplay between histone modifications and transcription factors in regulating adipogenesis and thermogenesis (Figure 1).

#### 2.3.2. Histone Modifications and Obesity-Related Metabolism

Histone modifications are an indispensable regulatory mechanism in energy metabolism, particularly through methylation, acetylation, phosphorylation, and ubiquitination. Concerning methylation, jumonji domain-containing protein 3 (JMJD3), as a histone demethylase, can demethylate H3K27me3. Upon fasting-activated PKA signaling, JMJD3, together with SIRT1 and PPARα, epigenetically activates β-oxidation genes to maintain energy balance [170]. Another JmjC domain-containing demethylase, jumonji histone demethylase 2a (JHDM2A), directly binds to the PPAR responsive element (PPRE) of the *Ucp1* gene. It is noteworthy that β-Adrenergic activation-induced binding of Jhdm2a to the PPRE of the *Ucp1* gene not only reduces the dimethylation of H3K9 (H3K9me2) at the PPRE but also facilitates the recruitment of transcription factors (*Rxrα* and *Pparγ*) and their co-activators (SRC1, CBP/P300, and PGC-1α) to the PPRE, thus enhancing *Ucp1* expression and regulating fatty acid oxidation and thermogenesis in lipid metabolism [171]. In the context of acetylation, male absence on the first (MOF) acts as a lysine acetyltransferase to participate in the acetylation of lysine 16 on histone H4 (H4K16ac) [172]. MOF-mediated H4K16ac regulates glucose uptake and lipid storage in adipocytes by controlling the expression of *Pparg* and its entire downstream transcriptional network [173]. Moreover, deletion of HDAC11 activates the adiponectin-adiponectin receptor (AdipoR)-AMPK pathway in the liver, which effectively alleviates obesity-related metabolic diseases [174]. Phosphorylation of histones also contributes to metabolic regulation. Carbohydrate response element binding protein (ChREBP) binds to the carbohydrate response element (ChoRE) to upregulate FASN expression through histone acetylation, methylation, and phosphorylation of histone H3 at serine 10 (H3S10). In contrast, protein phosphatase 2A (PP2A) counteracts PKA by dephosphorylating histone H3 serine 28 phosphorylation (H3S28ph), thereby repressing transcription and reducing the expression of gluconeogenic genes. With regard to ubiquitination, ring finger protein 20 (RNF20), an E3 ligase critical for the monoubiquitination of histone H2B at lysine 120 (H2Bub), is essential for adipose tissue homeostasis, as its adipocyte-specific deletion in mice results in progressive fat loss, organomegaly, and hyperinsulinemia [175]. Mechanistically, *Rnf20* knockdown in adipocytes reduces H3K4me3 occupancy at the solute carrier family 2 member 4 (*Slc2a4*) gene locus, thus suppressing glucose transporter type 4 (GLUT4) expression [176].

In addition to these classical modifications, emerging modifications such as histone lactylation and palmitoylation have recently garnered increasing attention. Histone lactylation, derived from intracellular lactate [177], has been shown to impact various metabolic processes. The genome-wide localization of histone H3 lysine 18 lactylation (H3K18la) was investigated in a broad panel of in vitro and in vivo samples, revealing that histone lactylation is present in tissues representing diverse metabolic states, with H3K18la marking active promoters [178]. Notably, Lactate-induced histone H4 lysine 12 lactylation (H4K12la) enhances the transcriptional level of peptidylglycine α-amidating monooxygenase (*Pam*), thereby promoting the synthesis of α-melanocyte-stimulating hormone (α-MSH), which in turn suppresses appetite and improves metabolic homeostasis [179]. Similarly, recent findings have revealed a regulatory role of palmitoylation in metabolic processes. The zinc finger DHHC-type palmitoyltransferase 23 (ZDHHC23) catalyzes the palmitoylation of plant homeodomain finger protein 2 (PHF2), thereby facilitating its ubiquitin-dependent degradation. Since PHF2 directly destabilizes SREBP-1C, this modification ultimately suppresses SREBP-1C-dependent lipogenesis [180]. Although palmitoylation has been identified on histone H3 and H4 [181,182], its specific role in adipocyte-related epigenetic regulation remains to be investigated.

#### 2.3.3. Histone Modifications in the Adipose Tissue Microenvironment

Histone modifications regulate the adipose tissue microenvironment through multiple mechanisms. The acute treatment of hepatocytes with lipopolysaccharide (LPS) induces increased histone acetylation at H3K9 and lysine 18 (H3K9ac and H3K18ac) at the loci of inflammatory genes such as *Tnf-α* and chemokine (C-C motif) ligand 2 (*Ccl2*) [183]. Moreover, obese children with IR exhibit reduced expression levels of both SIRT1 and SIRT2 in PBMCs [184]. In addition, the loss of HDAC6 alters the composition of the gut microbiota, characterized by an increase in *Bacteroides* and *Parabacteroides* and a decrease in the S24-7 family and *Lactobacillus*. These alterations may exacerbate obesity by impairing the functionality of regulatory T cells [185]. Overall, histone modifications shape the inflammatory and metabolic landscape of obese adipose tissue.

### 2.4. Additional Epigenetic Mechanisms Implicated in Obesity

#### 2.4.1. Non-Coding RNAs

Non-coding RNAs (ncRNAs) are RNA molecules that do not code for proteins, but instead play vital regulatory roles in gene expression [186,187,188]. Among them, microRNAs (miRNAs), long non-coding RNAs (lncRNAs), and circular RNAs (circRNAs) have emerged as key players in the regulation of adipose tissue development. In morbidly obese adolescents, at least ten circulating miRNAs show strong associations with BMI, waist-to-height ratio (WHtR), and adipokine levels [189]; among them, miR-122 and miR-34a are upregulated in children with obesity accompanied by non-alcoholic fatty liver disease (NAFLD) and/or IR [190], and miR-486 is implicated in accelerating preadipocyte proliferation [191]. Furthermore, miR-27 has been shown to inhibit adipocyte differentiation by targeting PPARγ [192], miR-375 prevents HFD–induced obesity by targeting the aryl hydrocarbon receptor and the bacterial tryptophanase gene [193], whereas miR-22 drives thermogenic function in BAT by regulating glycolysis and thermogenic genes via the hypoxia-inducible factor 1α (*HIF1α*) and mechanistic target of rapamycin complex 1 (mTORC1) signaling pathways [194]. CircRNAs modulate gene expression indirectly by acting as miRNA sponges. CircSAMD4A regulates preadipocyte differentiation by acting as a miR-138-5p sponge, and thus increasing enhancer of zeste homolog 2 (EZH2) expression [195]. EZH2, a histone methyltransferase targeting H3K27, directly represses *Wnt* genes to promote adipogenesis [196]. In contrast, the knockdown of circRNA CDR1as promotes osteogenesis and suppresses adipogenesis of bone marrow-derived mesenchymal stem cells (BMSCs) via the circCDR1as-miR-7-5p-WNT family member 5B (WNT5B) axis [197]. In addition, several lncRNAs exert profound regulatory effects on adipogenesis and thermogenesis. MSTRG4710 promotes adipogenesis by upregulating PPARγ and C/EBPα expression, accompanied by an increased number of lipid droplets [198]. Through transcriptome assembly and meta-analysis of public datasets, several lncRNAs specifically expressed in BAT and WAT have been identified [199,200]. Among these, zinc finger and BTB domain-containing protein 7b (ZBTB7B), a transcriptional factor, is essential for the differentiation of brown and beige adipocytes in vitro. Functionally, ZBTB7B promotes thermogenic gene expression by recruiting the heterogeneous nuclear ribonucleoprotein U (hnRNPU)/Blnc1 ribonucleoprotein complex, thereby enhancing pathways related to fuel oxidation and thermogenesis [201]. Another lncRNA, XIST, is expressed at significantly higher levels in female than in male adipose tissues; its knockdown impairs the differentiation of brown preadipocytes, at least in part by disrupting its interaction with C/EBPα [202]. Moreover, lncRNA βFaar inhibits lipid droplet swelling by binding to Ras-related protein Rab-18 (RAB18) and promoting interferon regulatory factor 4 (IRF4) nuclear translocation, increases *Ucp1* transcription, and further induces inguinal WAT browning by binding to karyopherin subunit alpha 6 (KPNA6) [203]. Together, these findings highlight the intricate involvement of ncRNAs in orchestrating adipocyte fate decisions through epigenetic and post-transcriptional regulation.

Growing evidence indicates that ncRNAs play crucial regulatory roles in energy metabolism [204,205]. Numerous studies have identified distinct miRNAs associated with metabolic regulation and NAFLD pathogenesis, including hsa-MIR-6236 linked to insulin sensitivity [206], miR-32-5p and miR-339-3p correlated with adipose tissue macrophage signatures [207], and miR-122-5p, miR-1343-5p, miR-193a-5p, miR-193b-5p, and miR-7845-5p positively associated with key histological features of NAFLD [208]. Among these, miR-802 inhibits hepatic AMPK expression by binding to the 3′UTR of mouse protein kinase, AMP-activated, beta 1 non-catalytic subunit (*Prkab1*) or human protein kinase AMP-activated catalytic subunit alpha 1 (*PRKAA1)*, disrupting metabolic homeostasis [209], while miR-690 regulates macrophage inflammatory responses and insulin signaling through targeting nicotinamide adenine dinucleotide kinase (*Nadk*) [210]. Furthermore, circRNF111 sponges miR-143-3p, activating the insulin-like growth factor 2 receptor (IGF2R)-mediated insulin signaling pathway (IRS1–PI3K–AKT) and ultimately promoting glucose uptake while inhibiting lipid synthesis [211]. LncRNAs are also involved in metabolic regulation. A negative correlation between lncRNA HOTAIR expression in subcutaneous fat depots from the arm or abdomen and regional adiposity has been observed in individuals with severe obesity or uremia [212]. Conversely, in hepatic tissue, HOTAIR is significantly upregulated in patients with type 2 diabetes mellitus and mice fed with an HFD [213]. These findings suggest that ncRNAs may serve as potential therapeutic targets for metabolic diseases such as obesity, although their regulatory mechanisms require further investigation in a tissue- and context-specific manner.

#### 2.4.2. Chromatin Remodeling

Chromatin remodeling represents a critical epigenetic mechanism that regulates gene expression by altering the structure and accessibility of chromatin to transcription factors [214]. ATP-dependent chromatin remodeling complexes, such as switch/sucrose nonfermentable (SWI/SNF) and inositol requiring 80 (INO80), alter nucleosome positioning to regulate numerous DNA-templated processes [215]. Notably, chromatin modification and remodeling genes are markedly enriched in tumor tissues of obese patients with prostate cancer compared to those from individuals with a healthy weight [216]. This observation suggests that chromatin dynamics may be influenced by obesity-related metabolic alterations in disease contexts. Consistently, chromatin remodeling complexes are also known to play essential roles in adipocyte differentiation and metabolic regulation. Dynamic changes in chromatin accessibility during forskolin (FSK)-induced cAMP stimulation induce adipocyte browning [217]. Among chromatin remodeling factors, BRG1/BRM-associated factor 60b (BAF60b) has been identified as a key chromatin-remodeling factor that modulates hepatic lipid metabolism and the progression of NAFLD by interacting with C/EBPβ to regulate PPARγ transcription [218]. In addition, myeloid-specific BAF60a knockout promotes adipose tissue macrophage pro-inflammatory activation, exacerbating diet-induced obesity, IR, and metabolic dysfunction [219].

The proliferative expansion of β-cells correlates with weight gain and the extent of IR in HFD-induced models [220,221]. Numerous chromatin remodeling complexes play critical roles in maintaining β-cell function in vivo. In particular, β-cell–specific inactivation of the SWI/SNF complex in mice impaired the ability of the pancreatic transcription factor pancreatic and duodenal homeobox 1 *(Pdx1)* to bind the insulin gene enhancer, thereby reducing insulin gene expression and ultimately disrupting whole-body glucose homeostasis [222]. Similarly, Chromodomain helicase DNA-binding protein 4 (CHD4), a subunit of the nucleosome remodeling and deacetylase (NuRD) complex, is required in mature β-cells, as its loss leads to glucose intolerance and impaired insulin secretion [223]. Further investigation is needed to clarify the roles of chromatin remodeling in obesity and metabolic regulation, especially its interactions with other epigenetic mechanisms.

#### 2.4.3. Post-Translational Modifications

Post-translational modifications (PTMs) such as phosphorylation, ubiquitination, acetylation, and lactylation critically influence adipocyte differentiation by modulating transcription factor activity. Among these, acetylation and phosphorylation regulate essential signaling cascades and transcriptional programs involved in adipogenesis [224,225]. PR domain-containing protein 16 (PRDM16) acetylation at lysine 915 disrupts its interaction with PPARγ, thereby inhibiting WAT browning [226]. HDAC6-mediated deacetylation of cell death-inducing dffa-like effector c (CIDEC) reduces its stability and impairs lipid droplet fusion [227]. Increased acetylation of SREBP-1c at lysines 289 and 309 by CBP and E1A binding protein p300 (p300) during hepatic lipogenesis upregulates lipogenic genes [228]. Phosphorylation of PPARγ at serine 273 promotes IR by upregulating growth differentiation factor 3 (GDF3) in adipose tissue macrophages (ATMs) and skeletal muscle [229]. Furthermore, phosphorylation of AMPKα at serine 496 impairs AMPK-mitochondrial fission factor (MFF) signaling and disrupts mitochondrial dynamics [230]. SUMOylation, the covalent attachment of small ubiquitin-like modifier (SUMO) proteins, modulates adipogenic gene expression networks. Ubiquitin-specific protease 1 (USP1) promotes adipogenesis by deubiquitinating and stabilizing C/EBPβ [231]. Likewise, the knockdown of SUMO-specific protease 1 (*Senp1*) reduces the expression of key adipogenic regulators, which impairs the migratory and proliferative capacities of human adipose-derived stem cells, and ultimately inhibits adipocyte differentiation [232,233]. In contrast, SUMOylated C/EBPβ recruits death domain-associated protein (DAXX) to repress homeobox C10 (*HOXC10*), thereby promoting adipose tissue browning [234]. Additionally, Iroquois homeobox 3 (*Irx3*) acts as an upstream regulator of SUMOylation, and its repression of SUMOylation is identified as a key mediator of its pro-adipogenic effects [235]. Beyond these direct effects, PTMs also interact with other epigenetic mechanisms to influence chromatin structure and transcriptional regulation in obesity. Notably, SUMOylation may contribute to the stable silencing of pre-adipocyte-specific genes in mature adipocytes by promoting the recruitment or activation of chromatin repressors, such as polycomb group complexes and DNMTs [236,237,238].

PTMs are significant in regulating energy metabolism by modifying key enzymes or proteins [239]. In mouse hepatocytes, increased acetylation of phosphoenolpyruvate carboxykinase (PEPCK) blocks its proteasomal degradation when accompanied by decreased ubiquitination, thus enhancing hepatic gluconeogenesis [240]. Conversely, under normal conditions, acetylation of PEPCK promotes its interaction with E3 ubiquitin ligases, leading to proteasome-dependent degradation and suppression of glucose production [241]. Phosphorylated succinyl-CoA synthetase (SUCLA2) in ATMs is negatively associated with obesity in humans, suggesting a regulatory role in metabolic balance. Phosphorylation also modulates glucose and lipid metabolism. AMPK and forkhead box O1 (FOXO1) phosphorylation suppresses hepatic gluconeogenesis by downregulating key enzymes such as PEPCK and glucose-6-phosphatase (G6PASE) [242], while mTOR-mediated phosphorylation of CRTC2 relieves its inhibition of coat protein complex II (COPII)-dependent SREBP1 maturation to modulate hepatic lipid metabolism [243]. SUMOylation plays a parallel role in metabolic regulation. Previous studies have shown that cytokine stimulation induces endoplasmic reticulum stress, accompanied by alterations in the SUMOylation profile in mouse and human pancreatic β-cells [244]. The loss of SUMOylation at lysine 76 of endoplasmic reticulum protein 44 (ERP44) enhances its degradation and disrupts binding to endoplasmic reticulum oxidoreductase 1 alpha (ERO1A), protecting mice from HFD-induced metabolic disorders [245]. Additionally, SUMO-specific protease 2 (SENP2)-mediated deSUMOylation of PPARα promotes its degradation, suppressing FGF21 expression and fatty acid oxidation [246]. Lactylation, a newly recognized PTM, is elevated in skeletal muscle of obese females and correlates with IR [247]. In the liver, lactylation at lysine 673 of FASN suppresses its enzymatic activity, thereby reducing hepatic lipid accumulation via mitochondrial pyruvate carrier 1 (MPC1)-mediated pathways [248]. Collectively, these diverse PTMs finely regulate adipogenesis and metabolic homeostasis.

## 3. Conclusions and Future Perspectives

Recent advances in epigenetic research have significantly deepened our understanding of obesity pathogenesis. Specifically, epigenetic modifications regulate obesity-associated gene expression by altering chromatin accessibility, recruiting transcriptional regulators, and shaping complex regulatory networks, ultimately influencing adipocyte differentiation and metabolic homeostasis. About DNA methylation, studies have not only pinpointed the CpG sites associated with obesity but also clarified the regulatory mechanisms of DNMTs from multiple dimensions. Regarding RNA methylation, m^6^A indicates the role of various key enzymes and related proteins in the obesity process. In histone modifications, several key enzymes have been identified as regulators of lipid metabolic reprogramming and insulin signal transduction. In addition, non-coding RNAs, chromatin remodeling complexes, and post-translational modifications act as crucial epigenetic regulators in obesity by modulating adipocyte differentiation, metabolic pathways, and gene expression through distinct layers of epigenetic regulation.

Epigenetic mechanisms interact in various ways to establish a complex regulatory network, exerting multidimensional control over cellular metabolism. During the BAT-myocyte remodeling process, the histone demethylase ubiquitously transcribed tetratricopeptide repeat, X chromosome-encoded (UTX) reduces H3K27me3 levels in the promoter of *Prdm16*, thus promoting its high expression. PRDM16 subsequently recruits DNMT1 to the myogenic differentiation 1 (*Myod1*) promoter, inducing hypermethylation of the *Myod1* promoter and inhibiting *Myod1* expression. This process supports the maintenance of the brown adipocyte character and prevents its transition into myocytes [249]. Additionally, in the DNMT1-mediated METTL16/m^6^A-YTHDC2/SCD1 axis, DNA and RNA methylation cooperate to regulate lipid metabolism [250]. Moreover, RNA methylation influences the function of lncRNAs in adipogenesis; METTL14 enhances the m^6^A modification of LINC00278, facilitating its interaction with brahma-related gene 1 (BRG1) and subsequent activation of the PPARγ2 pathway [251]. Furthermore, RNA modification and protein phosphorylation jointly contribute to an integrated regulatory axis that governs metabolic homeostasis and tumor progression, as exemplified by the ALKBH5–GCGR/mTORC1, FTO–PPARγ/AMPK, and METTL3-large tumor suppressor kinase 1 (LATS1)/Hippo signaling pathways [131,252,253]. Despite these advances, the interactions between epigenetic mechanisms in obesity and related metabolic diseases are still not clear and require more research to provide a basis for clinical treatment and prevention.

Interestingly, epigenetic mechanisms not only affect adipogenesis and its associated metabolism but also influence the maintenance of weight loss through epigenetic memory. Current studies suggest that obesity-induced alterations in histone modifications (H3K27me3, H3K4me3) and chromatin accessibility in adipocytes do not fully revert to normal levels after weight loss. When adipocytes are re-exposed to an obesogenic environment, these persistent epigenetic marks facilitate the reactivation of genes involved in inflammation and metabolic dysregulation [254], which is a key factor in weight regain. In addition, the transgenerational epigenetics of obesity has garnered increasing attention. Maternal obesity can induce epigenetic modifications like DNA methylation in oocytes, which are transmitted to subsequent generations and influence the risk of metabolic disorders in offspring [255]. Nevertheless, the underlying mechanisms of these effects remain unclear.

Research on the epigenetics of obesity still has limitations. (1) Most animal experiments only focus on a single tissue, such as the liver or adipose tissue, lacking a synchronous epigenetic analysis across multiple tissues; (2) future research should aim to delineate the heterogeneity of epigenetic modifications within and across metabolic tissues; (3) the synergistic or antagonistic effects among epigenetic modifications in the regulation of obesity occurrence remain unclear and require further exploration; (4) integrating longitudinal human cohort studies with multi-omics approaches is essential for elucidating the role of epigenetic alterations in obesity; and (5) translating these epigenetic findings into clinical applications poses significant challenges.

## Figures and Tables

**Table 1:** The link between DNA methylation and obesity.

Tissue	Subjects	Demographics	Affected Gene	Result	References
Peripheral blood	Individuals with obesity (*n* = 67; BMI > 25)	Japanese, Male and Female	*Fgf21* (CpG sites)	At least 5 obesity-related CpG sites are identified on the *Fgf21*	[31]
Placenta and umbilical cord	Mother–infant pairs (*n* = 114)	Spanish, Female	*Hadha* (CpG sites), *Slc2a8* (CpG sites;)	CpG sites associated with pre-pregnancy BMI: 1031 in placenta; 369 in umbilical cord	[32]
Peripheral blood	Obese group (*n* = 41) Normal group (*n* = 31)	Chinese, Male and Female	*Tfam* (cg05831083), *Piezo1* (cg14926485)	CpG sites cg05831083 and cg14926485 are linked to childhood obesity	[33]
Adipocytes in subcutaneous and visceral adipose tissue	Patients with severe obesity and healthy controls (*n* = 190)	Multi-ethnic, Male and Female	*Prrc2a* (CpG sites in intronic enhancer regions), *Limd2* (CpG sites in proximal enhancer and exons)	5mC sites associated with obesity: 691 in subcutaneous; 173 in visceral	[21]
Liver	Individuals with obesity (*n* = 51)	Finnish, Male and Female	*Prkca* (CpG sites), *Tspo* (CpG sites)	3169 CpG sites linked to liver saturated fat content in obesity.	[34]
Blood	Obese group (*n* = 4; BMI > 30) Normal group (*n* = 4; BMI < 25)	Chinese, Male and Female	*Inhbb*, *Hoxa9*, *Tnnt3*, *Crtc1*, *Zbtb7b* (all CpG sites)	5 key methylated genes and 5 related signaling pathways identified in obese patients	[20]
Blood	Female with obesity (*n* = 13)	Brazilian, Female	*Pik3r1* (CpG sites in promoter and 5′UTR regions)	6 differentially methylated CpG sites in *Pik3r1*	[23]
Peripheral blood leukocyte	Female with obesity (*n* = 11)	Multi-ethnic, Female	Genes in MAPK, cAMP, and PI3K-Akt pathways	16,064 CpG (9236 genes) sites changed	[22]
Peripheral blood	Lean group (*n* = 60; BMI < 25) and obese group (*n* = 60; BMI > 30)	Norwegian, Female	Genes in obesity-related pathways (*Sbno2*, *Rps6ka2*, *Dmap1*, *Socs3*, *Setbp1*)	10 differentially methylated CpG sites linked to 8 gene loci and an enhancer locus at chromosome 2 were found	[35]
Peripheral blood	Adolescents (*n* = 263)	Multi-ethnic, Male and Female	*Sim1* (CpG sites)	5669 CpG sites associated with BMI percentile, 28 within obesity-related genes	[36]
Peripheral blood	Adults (*n* = 474)	Multi-ethnic, Male and Female	Genes involved in longevity-regulating pathways (*Mtor*, *Ulk1*, *Adcy6*, *Igf1r*, *Creb5*, *Rela*)	6 of these CpG sites, located at *Mtor* (cg08862778), *Ulk1* (cg07199894), *Adcy6* (cg11658986), *Igf1r* (cg01284192), *Creb5* (cg11301281), and *Rela* (cg08128650), were common to the metabolic traits	[37]
Sperm	Obese group (*n* = 20) Normal group (*n* = 47)	Multi-ethnic, Male	*Tp53aip1*, *Spata21*, *Soga1*, *Adam15* (all CpG sites)	3264 CpG sites associated with BMI were enriched in genes involved in key regulatory pathways	[38]
Saliva	Children (*n* = 75)	Hispanic, Male and Female	*Nrf1* (cg01307483)	Nrf1 is associated with obesity in 36-month-old children	[39]
Blood	Female with obesity (*n* = 18)	Japanese, Female	*Fto* (CpG1, CpG3, total CpG)	Weight loss reduces visceral fat and increases CpG3 methylation	[40]

The columns represent the following: Tissue, the type of biological sample used for methylation analysis; Subjects, description of the study population; Demographics, ethnicity and sex of the study participants; Affected Gene, specific genes found to be associated with obesity through differential methylation; Result, summary of the key findings; References, citation number corresponding to each study, as listed in the reference section. Abbreviations: CpG site, cytosine-phosphate-guanine; 5mC, 5-methylcytosine; BMI, body mass index; 5′UTR, 5′ untranslated region; MAPK, mitogen-activated protein kinase; cAMP, cyclic adenosine monophosphate; PI3K-Akt, phosphoinositide 3-kinase–AKT; *Fgf21*, fibroblast growth factor 21; *Hadha*, hydroxyacyl-CoA dehydrogenase trifunctional multienzyme complex subunit α; *Slc2a8*, solute carrier family 2 member 8; *Tfam*, transcription factor A, mitochondrial; *Piezo1*, piezo-type mechanosensitive ion channel component 1; *Prrc2a*, proline-rich coiled-coil 2A; *Limd2*, LIM domain containing 2; *Prkca*, protein kinase C α; *Tspo*, translocator protein; *Inhbb*, inhibin subunit beta B; *Hoxa9*, homeobox A9; *Tnnt3*, troponin T3, fast skeletal type; *Crtc1*, CREB-regulated transcription coactivator 1; *Zbtb7b*, zinc finger and BTB domain containing 7B; *Pik3r1*, phosphoinositide-3-kinase regulatory subunit 1; *Sbno2*, strawberry notch homolog 2; *Rps6ka2*, ribosomal protein S6 kinase A2; *Dmap1*, DNA methyltransferase 1-associated protein 1; *Socs3*, suppressor of cytokine signaling 3; *Setbp1*, SET binding protein 1; *Sim1*, single-minded family bHLH transcription factor 1; *Mtor*, mechanistic target of rapamycin kinase; *Ulk1*, UNC-51 like autophagy activating kinase 1; *Adcy6*, adenylate cyclase 6; *Igf1r*, insulin-like growth factor 1 receptor; *Creb5*, cAMP-responsive element-binding protein 5; *Rela*, RELA proto-oncogene, NF-κB subunit; *Tp53aip1*, tumor protein p53-regulated apoptosis-inducing protein 1; *Spata21*, spermatogenesis associated 21; *Soga1*, suppressor of glucose, autophagy associated 1; *Adam15*, ADAM metallopeptidase domain 15; *Nrf1*, nuclear respiratory factor 1; *Fto*, fat mass and obesity-associated protein.

## Abbreviations

The following abbreviations are used in this manuscript:

**Abbreviation**	**Full Name**
4e-bp1	4E-binding protein 1
5mC	5-methylcytosine
ALYREF	Aly/REF export factor
AMPK	AMP-activated protein kinase
Atg5	Autophagy-related 5
Bmp8b	Bone morphogenetic protein 8b
C/EBPα	CCAAT/enhancer binding protein α
CCNA2	Cyclin A2
CDK2	Cyclin-dependent kinase 2
Cdkn1a	Cyclin-Dependent Kinase Inhibitor 1A
DMPs	Differentially methylated positions
DNMTs	DNA methyltransferases
EMT	Epithelial–mesenchymal transition
EP3	Prostaglandin E receptor 3
FTO	Fat mass and obesity-associated
Fasn	Fatty acid synthase
FGF19	Fibroblast growth factor 19
H4K16ac	Acetylation of lysine 16 on histone H4
H3K9me2	Dimethylation of histone H3 at lysine 9
HDAC1	Histone deacetylase 1
IGF2BPs	Insulin-like growth factor 2 mRNA-binding proteins
INSR	Insulin receptor
Jak2	Janus kinase 2
JMJD3	Jumonji domain-containing protein 3
JHDM2A	Jumonji histone demethylase 2a
Klf9	Krüppel-like factor 9
LSD1	Lysine-specific demethylase 1
m^6^A	N6-methyladenosine
MOF	Male absence on the first
METTL	Methyltransferase-like
MBPs	Methyl-binding proteins
Myod1	Myogenic differentiation 1
NADP	Nicotinamide adenine dinucleotide phosphate
NSUN2	NOP2/Sun domain family, member 2
Nsd2	Nuclear receptor-binding SET domain 2
PPARγ	Peroxisome proliferator-activated receptor γ
PGC1α	Peroxisome proliferator-activated receptor-γ coactivator 1α
Pik3r1	Phosphoinositide-3-kinase regulatory subunit 1
Prdm16	PR domain-containing protein 16
PRMT1	Protein arginine N-methyltransferase 1
PKA	Protein kinase A
Srebp-1c	Sterol regulatory element-binding protein 1c
SIRT1	Silent mating-type information regulation 2 homolog 1
STAT3	Sgnal transducer and activator of transcription 3
SMURF2	Smad ubiquitination regulatory factor 2
SUV420H2	Suppressor of variegation 4-20 homolog 2
TNF-α	Tumor necrosis factor α
TET1	Ten-eleven translocation 1
UTX	Ubiquitously transcribed tetratricopeptide repeat, X chromosome-encoded
UCP1	Uncoupling protein 1
VASPIN	Visceral adipose tissue-derived serine protease inhibitor
VIRMA/KIAA1429	Vir-like m^6^A methyltransferase associated
WAT	White adipose tissue
WTAP	Wilms tumor 1-associated protein
Wnt10a	Wingless-type MMTV integration site family, member 10a
YTHDF1	YTH domain-containing family protein 1
Zfp410	Zinc finger protein 410
